# Estimation of seroprevalence of melioidosis among adult high risk groups in Southeastern India by indirect Hemagglutination assay

**DOI:** 10.1371/journal.pgph.0000431

**Published:** 2022-05-09

**Authors:** Sruthi Raj, Sujatha Sistla, Deepthy Melepurakkal Sadanandan, Tamilarasu Kadhiravan, Palanivel Chinnakali

**Affiliations:** 1 Department of Microbiology, JIPMER, Puducherry, India; 2 Department of Biostatistics, JIPMER, Puducherry, India; 3 Department of Medicine, JIPMER, Puducherry, India; 4 Department of Preventive and Social Medicine, JIPMER, Puducherry, India; Universidad Peruana Cayetano Heredia, PERU

## Abstract

*Burkholderia pseudomallei* is an environmental saprophyte known to cause melioidosis, a disease endemic in northern Australia and Southeast Asia. With the increasing number of melioidosis cases, there is a lack of data on seroprevalence rates and extent of exposure in high risk population of melioidosis from different endemic regions in India. The present cross sectional study was undertaken to estimate the seroprevalence of melioidosis in high risk populations in and around Puducherry, a coastal town in Southeastern India. Blood samples were collected from 275 diabetic individuals attending a tertiary care centre in Southern India and 275 farmers residing under the rural field practice area of our hospital. The antibody levels were estimated using an Indirect Hemagglutination Assay. The overall seropositivity was found to be 19.8% with a titer ≥1:20. Farmers were 2.8 times more likely to be seropositive than non-farmers. Rates of seroprevalence among diabetic subjects were less compared to the non-diabetic individuals. The seropositivity rates in non-diabetic farmers were higher (56/203, 27.6%) compared to diabetic farmers (34/164, 20.7%). The lowest seropositivity was seen among diabetic non-farmers at 10.4%. Multivariable logistic regression analysis revealed domicile (adjusted odds ratio—aOR: 2.32, 95% Confidence interval—CI: 1.05, 5.13) and contact with animals (aOR: 1.89, 95% CI:1.04, 3.44) as significant predictors of seropositivity. None of the other socio-demographic factors including gender and age were significantly associated with seropositivity. This study demonstrates widespread exposure to *B*. *pseudomallei* among adults residing in and around Puducherry, including those engaged in non-farming occupations.

## Introduction

*Burkholderia pseudomallei*, the causative agent of melioidosis, is known to exist in soil and water in endemic regions of Southeast Asia and northern Australia [1]. Clinically, melioidosis presents with a wide spectrum of disease manifestations ranging from acute systemic infections to chronic localised forms, that mimic other infections and leads to misdiagnosis and treatment failure [1]. Melioidosis is acquired through inoculation, inhalation or aspiration and ingestion. Diabetes mellitus and agricultural activities are the two important risk factors for acquiring melioidosis [2]. A higher risk of exposure to *B*. *pseudomallei* is seen in rice farmers as they regularly come in contact with contaminated soil [3].

In India, an increasing number of melioidosis cases have been reported from different regions with the maximum number of cases being recognised from Karnataka and Tamil Nadu [4]. This need not necessarily exhibit the true picture of melioidosis in India due to lack of awareness among microbiologists and clinicians. Moreover, limited research and lack of access to well-equipped diagnostic laboratories add to the misdiagnosis of melioidosis cases [4]. India has the dubious distinction of being the diabetes capital of the world with a majority of the population residing in rural areas engaged in agricultural activities [1,4].

In the absence of disease, individuals exposed to *B*. *pseudomallei* present in soil and water become seropositive and develop antibodies to *B*. *pseudomallei*. However, these antibodies are non-protective [5,6]. Seroprevalence studies can be used to estimate the exposure to *B*. *pseudomallei* in a given geographic region. Several methods have been employed for this purpose including the indirect hemagglutination assay (IHA) using crude antigen derived from *B*. *pseudomallei* strains. From India, except for one study from Karnataka [3], no other seroprevalence studies have been reported. There is a lack of data in relation to seropositivity and high-risk population including diabetics and farmers. The seroprevalence of melioidosis is important especially in a coastal town like Puducherry since the number of melioidosis cases has been increasing over the years. As reported elsewhere, 34 melioidosis cases were found between January 2014 and December 2018, from a single institution in Puducherry [7]. During the same period, 31 cases were identified from another center in the same region [8]. Therefore, we carried out an indirect hemagglutination test, to estimate the seroprevalence of melioidosis among the two high-risk populations in and around Puducherry.

## Materials and methods

### Study population and source of sera

A cross-sectional study was conducted from January 2020 to January 2021. The sample size was estimated with an anticipated prevalence of 29% (Manipal study) [3], an alpha error of 5% (95% confidence level) and absolute precision of 6%, the required sample size was estimated to be 220. Considering non-response to blood sample collection (approx.20%), the minimum sample size was estimated to be 275.

A total of 550 adults between 18 and 90 years of age, were enrolled in the study. The 275 adults who had attended the diabetic clinic, in a tertiary care center in Southern India were selected using consecutive sampling. For the remaining 275 adults involved in agricultural work and residing under the rural field practice area of our hospital (JIRHC, Ramanathapuram), one of the four villages under Ramanathapuram was randomly selected Convenient sampling was performed using a house-to-house survey and farmers were recruited based on their willingness to participate in the study. Two ml of blood sample was collected using venipuncture. Details of the study subjects such as their age, gender, occupation, area of residence, personal habits such as alcohol consumption and other socio-economic details were recorded in a structured proforma by a trained personal. Residence was characterised as urban if they were located within a metropolitan district or as a main city and as rural if they resided outside these areas [6]. Serum samples of the subjects were stored at -80°C until tested. IHA was performed as described previously [9]. Crude antigens were prepared in our laboratory from two clinical isolates of *B*. *pseudomallei*. Positive control was prepared using pooled serum from three culture-proven melioidosis cases with the titer of ≥1:10240. In the present study, chick erythrocytes were used instead of sheep red blood cells, as they are heavier (being nucleated) and tend to settle down faster during the agglutination reaction. Fresh chick erythrocytes were used in the assay without Double-aldehyde stabilisation (DAS). The optimal antigen dilution was determined against the known positive serum. Each sample was tested in triplicate to validate the test and an average was taken. A titer of ≥1:20 with agglutination of erythrocytes was considered positive [3].

### Ethics statement

This study was approved by the Institutional Ethics Committee for Human Studies, JIPMER, Puducherry (JIP/IEC/2018/0230) and written informed consent for all procedures was obtained from the participants.

### Statistical analysis

All statistical analyses were performed using SPSS software version 19.0 (IBM; Armonk, NY, USA). All the categorical variables were summarised as frequency and percentages. The age which does not follow normality was summarised as median with interquartile range. The normality assumption was checked using the Kolmogrov-smirnov test. Mann-Whitney U test was used to compare the median age across the seropositivity status. An Independent student t test was carried out to compare the log transformed titer values across gender. Chi-square or Fisher’s exact tests were performed to find the association of seropositivity with strata and with possible exposure to soil among the diabetic non-farmers. With the use of Univariate logistic regression, association between seropositivity status and known risk factors were determined. The unadjusted odds ratios and the 95% confidence intervals (CIs) were estimated. The variables which were found to have a *p*-value <0.20 in univariate analyses were included in the multivariable logistic regression. The multivariable logistic regression analysis was performed and adjusted odds ratios along with their 95% CIs were reported. All the statistical analyses were carried out at 5% level of significance and a *p*-value less than 0.05 was considered to be statistically significant.

## Results

**Fig 1:**
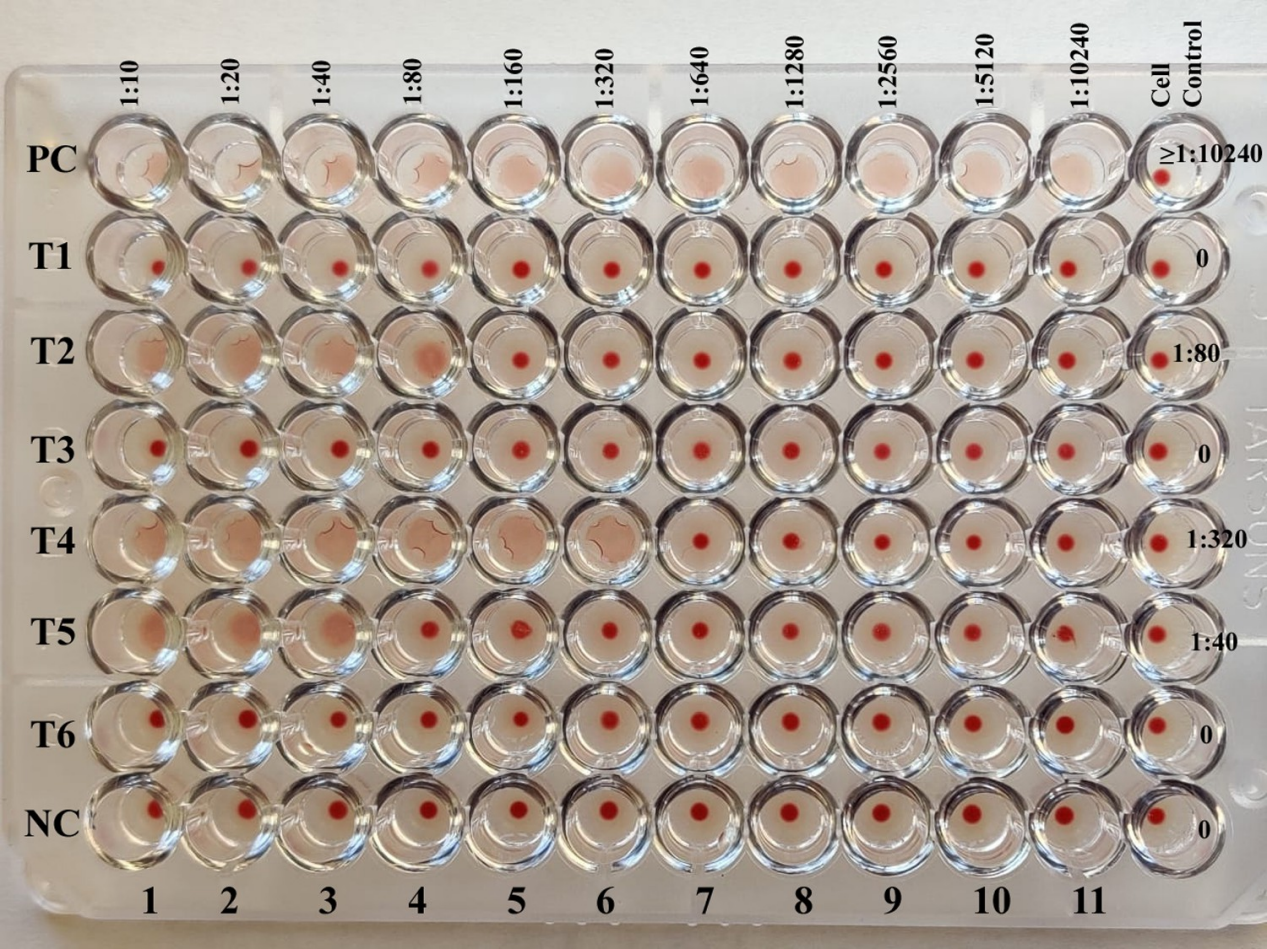
IHA plate with antibody titers. PC- positive control, NC**-** negative control, T1 -T6 are the test samples. Antibody titers are provided on the right.

**Fig 2:**
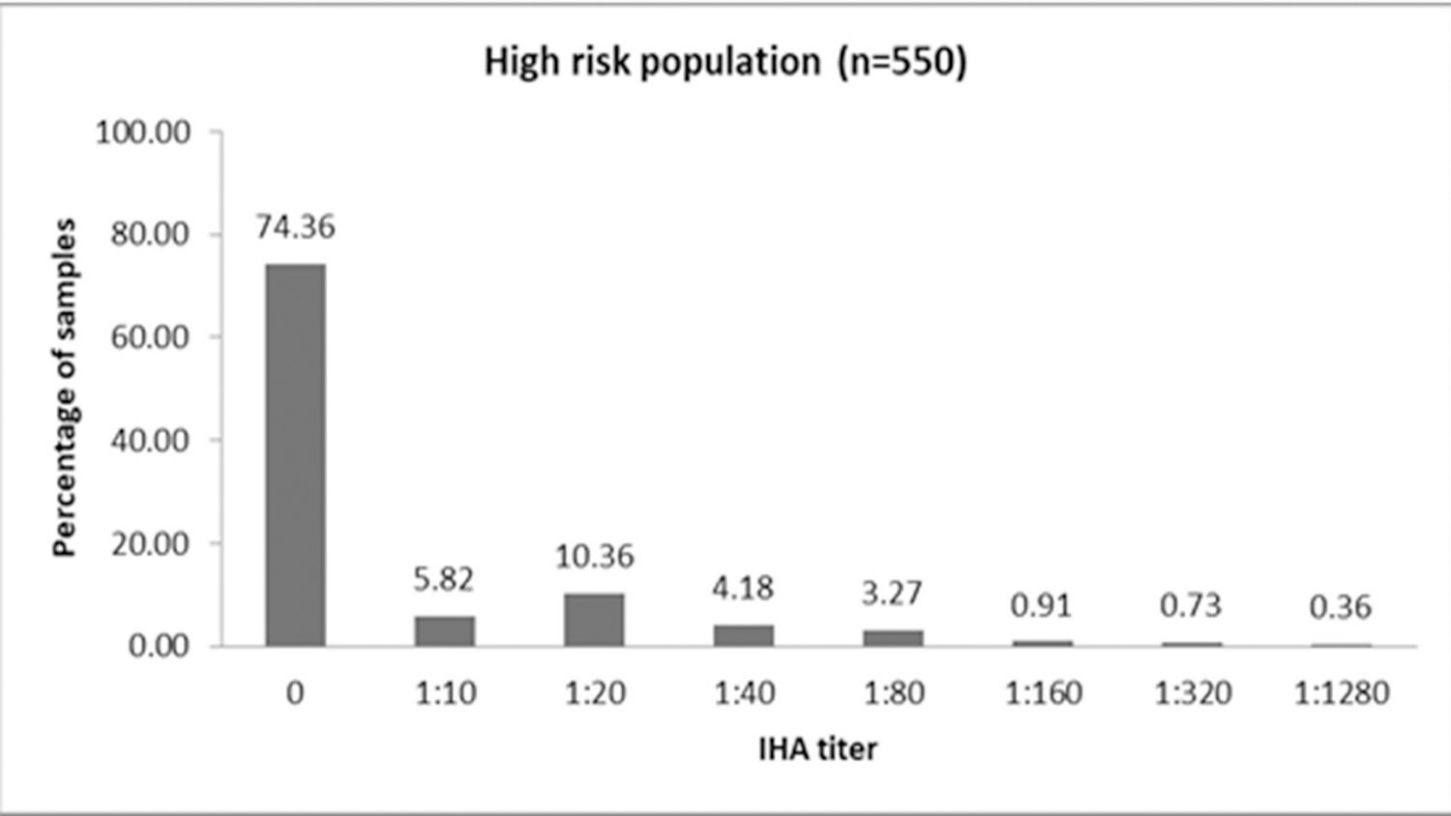
Distribution of IHA titers among high risk population.

The overall seroprevalence using antibody titers ≥ 1:20 was found to be 19.8% (95% CI: 13.72, 25.92). The picture of the IHA plate is depicted in (Fig 1). Out of 550 subjects enrolled in the study, there were 257 males and 293 females aged from 18 years to 90 years. The distribution of antibody titers among high risk population is shown in (Fig 2). The high risk population included 347 diabetic subjects and 367 farmers, as there were 72 diabetic subjects among 275 farmers and 92 farmers among 275 diabetic subjects. A total of 164 study subjects were diabetic farmers, 183 subjects were diabetic non-farmers and non-diabetic farmers were 203.

The median ages of seropositive and seronegative participants were found to be 55(IQR 44,65 years) and 56(IQR 47,63.5 years) respectively. Among the different age groups, seropositivity of 5.5% was found in the younger age group (18–30) compared to 56% in the elderly, aged ≥ 51 years. Seropositivity among the three different strata; diabetic farmers (n = 164), diabetic non-farmers (n = 183) and non-diabetic farmers (n = 203) were found to be 20.7% (95% CI:14.53–26.94), 10.4% (95% CI: 5.71,15.05) and 27.6% (95% CI: 20.75, 34.43) respectively. This result was found to be statistically significant (*p*<0.001). Among the diabetic non-farmers (n = 183), the majority of individuals 89.1% (163/183) were professionals (Government and private employees, bank employees, engineers, teachers, etc). Among the diabetic non-farmers, 10.0% (2/20) of subjects with possible exposure to soil were seropositive compared to 10.4% (17/163) of subjects who were unlikely to have occupational exposed to soil/water (p-value = 1.00).

An antibody titer of ≥1:10 was found in 32 additional subjects, which would increase the overall seropositivity to 25.6% (95% CI: 21.99, 29.29). Table 1 displays the results of the univariate logistic regression model for each risk factor, their associated seropositivity status, unadjusted odds ratios and its 95% confidence intervals. Seropositivity was not significantly associated with various factors such as gender, different age groups, smoking and alcohol intake, activities near water bodies, gardening and use of footwear. There was no significant difference between the geometric mean titre values among males 33.02 (95% CI:25.89, 42.12) and females 42.78 (95% CI:34.03, 53.77), (*p* = 0.14). Odds of seropositivity was 2.8 folds higher in farmers than non-farmers (OR:2.80,95% CI: 1.65–4.77, *p*<0.001). The odds of seropositivity was 2.85 folds higher in subjects who had contact with animals than who did not have animal contact (OR:2.85,95% CI: 1.77, 4.60, *p* < 0.001) and in rural dwellers odds of seropositivity was 3.69 folds higher than those who lived in urban districts (OR:3.69,95% CI:2.00, 6.81, *p* <0.001). However, the odds of having seropositivity was 53% lower in the diabetic group when compared to non-diabetic group (OR:0.47,95% CI: 0.31, 0.72, *p* = 0.001). Contact with animals and domicile were identified to be significant independent predictors through multivariable logistic regression analysis (Table 1). Rural dwellers had 2.32 folds higher odds of having seropositivity than those who lived in urban districts (aOR: 2.32, 95% CI:1.05, 5.13, *p* = 0.04) when adjusted for other variables in the model. The odds of having seropositivity was 1.89 folds higher in people who had contact with animals when compared to those who had no contact with animals when adjusted for other variables in the model. (aOR: 1.89, 95% CI: 1.04, 3.44, *p* = 0.04).

**Table 1:** Socio-demographic and clinical factors associated with seropositivity in high risk population.

Risk factors n = 550	Seropositivity^#^ (≥20) n(%) = 109 (19.8)*	Seronegativity (<20) n(%) = 441(80.2)*	Unadjusted OR (95% CI)	Adjusted OR^$^ (95% CI)
Gender Male (257) Female (293)	47(43.1) 62(56.9)	210(47.6) 231(52.4)	1.00 1.20 (0.79–1.83)	-
Age categories 18–30 (23) 31–40 (55) 41–50 (124) ≥51 (348)	6 (5.5) 13 (11.9) 29 (26.6) 61 (56.0)	17 (3.9) 42 (9.5) 95 (21.5) 287 (65.1)	1.00 0.88 (0.29, 2.69) 0.87 (0.31, 2.40) 0.60 (0.23, 1.59)	-
Diabetics Yes (347) No (203)	53 (48.6) 56 (51.4)	294 (66.7) 147 (33.3)	0.47 (0.31, 0.72) 1	0.82 (0.49, 1.37) 1
Occupation Farmers (367) Non-farmers (183)	90 (82.6) 19 (17.4)	277 (62.8) 164 (37.2)	2.80 (1.65, 4.77) 1	1.89 (0.78, 4.58) 1
Smoking Yes (86) No (464)	17 (15.6) 92 (84.4)	69 (15.6) 372 (84.4)	1.00 (0.56, 1.78) 1	-
Alcohol intake Yes (123) No (427)	26 (23.9) 83 (76.1)	97 (22.0) 344 (78.0)	1.11 (0.68, 1.82) 1	-
Activities near water bodies Yes (349) No (201)	76 (69.7) 33 (30.3)	273 (61.9) 168 (38.1)	1.42 (0.90, 2.23) 1	0.81 (0.48, 1.36) 1
Gardening Yes (382) No (168)	84 (77.1) 25 (22.9)	298 (67.6) 143 (32.4)	1.61 (0.99, 2.63) 1	0.55 (0.27, 1.11) 1
Wear footwear No(318) Yes (232)	72 (66.1) 37 (33.9)	246 (55.8) 195 (44.2)	1.54 (1.00, 2.39) 1	0.94 (0.55, 1.60) 1
Contact with animals Yes (316) No (234)	83 (76.1) 26 (23.9)	233 (52.8) 208 (47.2)	2.85 (1.77, 4.60) 1	**1.89 (1.04, 3.44)** 1
Domicile Rural (390) Urban (160)	96 (88.1) 13 (11.9)	294 (66.7) 147 (33.3)	3.69 (2.00, 6.81) 1	**2.32 (1.05, 5.13)** 1

^#^ Seropositive status based on antibody titer levels.

*Column percentage, CI: Confidence intervals, OR: Odds ratio.

^$^Adjusted odds ratio (OR) for a risk factor was adjusted for other variables in the model.

## Discussion

India is an endemic country for melioidosis with the successful isolation of *B*. *pseudomallei* from soil and water [1,10–12]. The present study provides new data involving a high risk population of diabetics and farmers, to determine the extent of exposure to *B*. *pseudomallei*. Overall seropositivity of 19.8% was noted in the study population. The IHA is a common serological test employed for seroepidemiological studies since it was first described in 1965, as it is simple, rapid and effective for the detection of antibodies [13,14]. However, IHA is also known for poor specificity and sensitivity due to high background seropositivity in melioidosis endemic countries [15]. In the present study a cut off titer of ≥ 1:20 was taken, to enhance the specificity of the test as described in a study from Southern India [3]. According to these authors, increasing the cut-off point may improve the specificity slightly, but it may affect the test sensitivity [16]. On the other hand, lowering the cut-off increases sensitivity as evidenced in the present study (seropositivity increased to 25.6% when a titer of ≥1:10 is taken as cut-off). The chick erythrocytes were not stabilised during the assay since fresh chick blood was used throughout the test, which prevented the lysis of RBCs. However, this did not affect the test as the positive well displayed loose button formations with ragged or folded edges (Fig 1).

The basic principle of IHA is the agglutination of erythrocytes, in the presence of serum antibodies to a variety of polysaccharide and lipopolysaccharide antigens, prepared using clinical isolates of *B*. *pseudomallei* [17]. Due to a wide degree of genetic diversity and antigenic variation, the use of pooled antigens from different clinical strains is preferred over a single strain [6]. The crude antigen used is a polysaccharide component, from the slime layer of the pathogen [18]. The sensitivity and specificity of IHA rely on concentrations and combinations of the crude antigens used in the assay [5]. There are speculations regarding cross-reacting antibodies to *B*. *thailandensis*, commonly found in soil along with *B*. *pseudomallei*.

The overall seropositivity was found to be 19.8% in the current study, which is comparable to the previous study reported from Southern India (29%) [3], and from endemic countries such as Thailand (38%) [6], Australia (3%) [19], Vietnam (6.4–31.8%) [20], Bangladesh (28.9%) [21]. These variations in seropositivity rates may be associated with the use of more sensitive serological techniques and the nature of crude antigens used in the IHA test [22]. Indirect enzyme-linked immunosorbent assay (ELISA) and complement fixation test are other serological tests used for serosurveillance studies [22,23]. ELISA was found to be the more sensitive method. However, the presence of non-specific cross-reactive antibodies that reacted with the crude antigen in indirect ELISA, resulted in a higher seropositivity rate in a study from Bangladesh [23]. Cross-reacting antibodies were also reported with the use of complement fixation test [22].

India has the world’s largest population diagnosed with diabetes and 25% of this population lives in rural settings with a predicted increase in diabetes from 51 million in 2010 to 87 million in 2030. [1,3]. Although diabetes is a major risk factor for acquiring melioidosis, no remarkable difference in seropositivity was found among diabetic individuals 48.6% (53/109) compared to non-diabetic individuals 51.4% (56/109). Similar results were obtained in a study from Karnataka [3]. Individuals with diabetes are known to be immunocompromised [6,17]. Hence, even after exposure the specific antibodies are produced at low levels and may not be detected in the IHA test.

A strong association is found between seropositivity and exposure to soil and water [24]. Farming is a major occupation in Southern India. Farmers are at a higher risk of acquiring melioidosis as their exposure to organisms present in the environment occurs repeatedly over a long period [2]. In the current study, we found farmers were significantly associated with seropositivity compared to non-farmers. Similar reports were found in farmers in Bangladesh [21]. In Thailand, farmers were 4.6 times more likely to be seropositive compared to non-farmers [6]. In contrast, no difference in seropositivity among farmers and non-farmers was reported from Karnataka [3] and Bangladesh [23]. According to a study from Thailand, diabetic farmers had a six to nine times higher risk of acquiring melioidosis compared to non-diabetic non-farmers, as impaired immunity in diabetes and high exposure in farmers act synergistically as risk factors to develop melioidosis [2]. In the present study, seropositivity in non-diabetic farmers was higher 27.6% (56/203) compared to diabetic farmers 20.7% (34/164). However, seropositivity was least among diabetic non-farmers 10.4% (19/183). This evidence further supports that farmers are highly exposed and are at higher risk of acquiring melioidosis. Even though diabetic farmers are equally exposed to contaminated soil and water, impaired immunity in diabetes lowers the detectable amount of antibodies to IHA and hence reduces their seropositivity rates. However, majority (89.1%) of individuals among the diabetic non-farmers were professionals where exposure to soil/water was unlikely, which explains the low seropositivity among them. However, no significant difference was observed in the seropositivity between the groups with possible and unlikely occupational exposure to soil/water. This lack of difference may be explained in two ways. One was the vast difference in the numbers (20 with occupational exposure vs 163 without occupational exposure) making comparison difficult. The second reason could be undocumented non occupational exposure to soil and water in the latter group.

Among other demographic factors studied, individuals living in rural areas and those having contact with animals were found to be significantly associated with seropositivity. Most of the residents in rural settings are farmers and they come in contact with animals at home and during work. They frequently get exposed to contaminated soil and water during agricultural activities and while helping animals graze the grasslands. This is further supported by a study from Thailand, where rural dwellers were three-fold more likely to be seropositive compared to the residents of urban districts [6]. Subjects from the urban districts lack exposure, moreover, a defective immune response is likely possible [25]. Also, over representation of rural dwellers in the study could lead to a lower comparative rate.None of the other personal habits such as smoking, alcohol intake, activities near water bodies, gardening and use of footwear were significantly associated with seropositivity in the present study. However, Vandana et al., in the Manipal study, reported gardening and activities near the river were positively associated with exposure [3]. Another study from Taiwan found that walking barefoot and flooding caused high risk of exposure [26].

The relationship between age and seropositivity is unclear. We found higher seropositivity rates in the elderly, aged ≥51 years compared to younger age groups which could reflect repeated exposure. A similar finding was reported from Bangladesh [23] where seropositivity of 30.4% was detected in patients over 50 years of age. In Thailand, seropositivity rates increased with advancing age in children [27–29]. On the other hand, an inverse relationship between seroprevalence rate and advancing age was found in Australia [30]. Another study from Southern India reported no significant differences in seropositivity rates in adults among different age groups [3]. However, IHA is considered to be more specific in young children compared to older age groups due to low background seroprevalence in neonates and young infants [25].

The study found no significant association of seropositivity with gender. There are conflicting reports of gender wise seropositivity rates with Australia [5] and Bangladesh [23] showing similar rates in both sexes while a study from southern India [3] reported a significantly higher seropositivity rate in females. Equal or higher seropositivity rates in females are contradictory to the prevalence of melioidosis which is far higher in males. According to Vandana et al., the possible reason for this could be fewer outdoor activities and exposure to low inoculum among females which might cause a seroconversion in the absence of disease, compared to males, who are likely to be exposed to a larger inoculum resulting in overt disease [3].

Globally, constant serosurveillance among high risk and rural populations is essential to monitor the pathogens that are of public health concern. As per the current study, seropositivity rates were higher among rice farmers. In a country like India where agriculture plays a vital role in the livelihood of most of the rural population, the farmers working in the fields could be recognised and monitored for the prevalence and incidence of melioidosis in their work location. Additionally, the current serosurveillance study provides useful information to plan and improvise targeted surveillance and to initiate the environmental control measures which could benefit the policymakers in the decision-making process. The public health professionals could focus on methods to create awareness among farmers about the risk of acquiring melioidosis and educate the population at risk.

The study had a few limitations. The human population surveyed in our study consisted of both diabetics and farmers who were at a higher risk of exposure to *B*. *pseudomallei*. The healthy adult population and children were excluded. Hence, seropositivity in non-diabetic non-farmers and for the younger age group could not be determined. Additionally, IHA used in the present study is known to have variable sensitivity and specificity with respect to the crude antigens used and the cross-reacting antibodies.

Further studies are required to determine seroprevalence using more sensitive and specific techniques and identify high risk populations for melioidosis from different regions in India. Along with this, environmental sampling for the presence of *B*. *pseudomallei* will help in estimating the true burden of melioidosis in India.

## Conclusions

This study has unequivocally demonstrated the evidence of exposure to *B*. *pseudomallei* in adults in and around Puducherry and strongly indicates that these individuals have the potential to develop melioidosis during their lifetime. There is a need for strengthening the laboratory facilities in rural areas. Besides, clinicians must consider melioidosis as a differential diagnosis in these exposed high risk populations to initiate early treatment for successful management of these cases. The observed seropositivity rates in high risk groups ascertain the usefulness of IHA as a promising diagnostic tool for seroepidemiological surveys.

## Supporting information

S1 Checklist STROBE statement—Checklist of items that should be included in reports of *cross-sectional studies*. (DOCX)

S1 Dataset (XLSX)
